# Idiopathic Anterior Scleritis in Pediatric Patients: A Report of Two Cases

**DOI:** 10.7759/cureus.73404

**Published:** 2024-11-10

**Authors:** José J López-Fontanet, Estefania Ramirez Marquez, Emilio Cepeda Terrasa, Sebastián J Vázquez-Folch, Armando Oliver, Carmen Santos

**Affiliations:** 1 Department of Ophthalmology, School of Medicine, Medical Sciences Campus, University of Puerto Rico, San Juan, PRI; 2 School of Medicine, Universidad Central del Caribe, Bayamón, PRI

**Keywords:** anterior scleritis, immunomodulatory therapy, intraocular inflammation, necrotizing scleritis, pediatric scleritis

## Abstract

This study presents two cases of idiopathic anterior scleritis in young female patients, one of whom progressed to necrotizing scleritis. Both cases posed significant diagnostic challenges and ultimately required immunomodulatory therapy after their respective inadequate responses to the initial standard treatments. Despite comprehensive laboratory evaluations, no underlying systemic etiology was identified in either patient. Pediatric anterior scleritis is extremely rare, and these cases underscore the importance of its early recognition and appropriate management to prevent sight-threatening and systemic complications.

## Introduction

Scleritis is an inflammatory condition of the sclera characterized by redness, swelling, and severe ocular pain [1]. The Watson system classifies scleritis into five categories based on its anatomical distribution: diffuse anterior, nodular anterior, necrotizing anterior without inflammation, necrotizing anterior with inflammation, and posterior scleritis [2,3].

Scleritis in pediatric patients is uncommon, with an incidence of approximately 1.2% [4]. Although posterior scleritis is more common in this population, anterior scleritis remains rare, with only a few cases reported in the literature [5]. The etiology of scleritis in children can be diverse, ranging from idiopathic causes to associations with systemic conditions, whether infectious or non-infectious. Infectious causes include bacterial, viral, fungal, and parasitic pathogens, while non-infectious causes are often related to autoimmune diseases such as juvenile idiopathic arthritis (JIA) and granulomatosis with polyangiitis [4,6].

Regardless of the extensive documentation of anterior scleritis in adults, the literature on its occurrence in the pediatric population is limited. The rarity of the condition in children presents challenges for diagnosis and management, necessitating further exploration. We report two children suffering from anterior scleritis.

## Case presentation

Case 1

An eight-year-old girl was referred to the clinic with a two-week history of left eye (OS) redness, swelling, and pain. She had flu-like symptoms around the same time, mainly consisting of headaches and a fever, both lasting 24 hours. The patient denied experiencing any associated diplopia, vision loss, or other ocular complaint. There was no notable past medical, familial, or ocular history. Her social history was significant only for her having multiple pets, including horses, roosters, unvaccinated cats, and vaccinated dogs. The review of systems was unremarkable.

Upon comprehensive ophthalmic evaluation, her best corrected visual acuity (BCVA) was 20/25 in both eyes (OU). Intraocular pressure with applanation was 15 mmHg in the right eye (OD) and 16 mmHg in the OS. The pupils were round and reactive to light, and there was no afferent pupillary defect. Her extraocular movements (EOMs) were full in all directions of gaze OU, without additional pain. The confrontation visual fields were full to count fingers in all quadrants OU. External and slit lamp examinations were normal in the OD. The OS presented fullness of the left upper eyelid, temporally over the lacrimal gland. There was superotemporal conjunctival injection as well as lacrimal gland erythema. The cornea, anterior chamber, iris, and lens were normal. A fundus examination showed normal retinas in OU.

**Figure 1:**
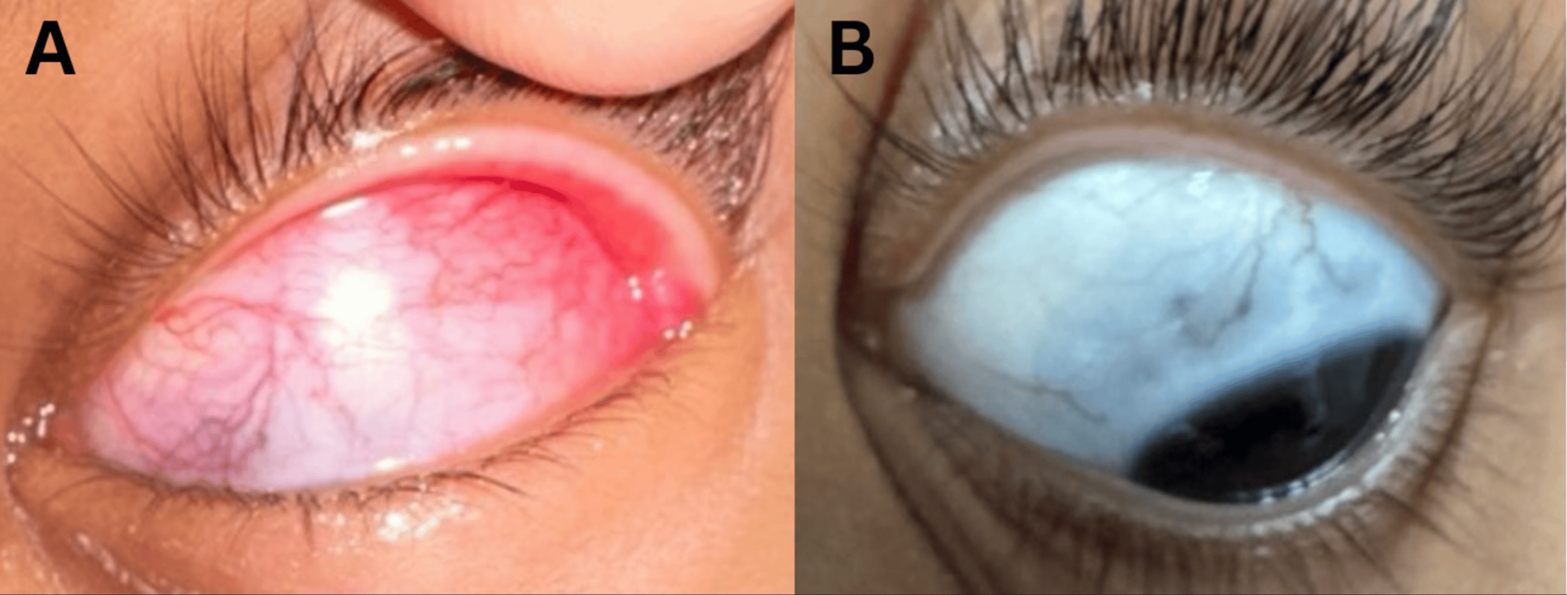
External photographs of the left eye. (A) Downward gaze showing scleral injection. (B) At the nine-month follow-up, leftward gaze shows a scleral bluish hue indicative of scleral thinning.

The patient was treated with prednisolone acetate 1% eye drops and systemic antibiotics for a presumptive diagnosis of viral dacryoadenitis. At the one-week follow-up, despite treatment, the patient presented with worsening upper eyelid inflammation, tenderness upon palpation, and a superior scleral injection that did not blanch with phenylephrine 2.5% in the OS (Figure 1A). She also complained of photophobia, painful EOMs, and left-sided headaches. These findings, particularly the non-blanching scleral injection and scleral tenderness, suggested a deeper inflammatory process involving the sclera, leading to a revised diagnosis of anterior scleritis. Given these findings, the patient was hospitalized for further workup and treatment. She was started on 20 mg of oral prednisone.

Magnetic resonance imaging (MRI) showed mild diffuse concentric wall thickening of the left eye globe with associated mild enhancement and mild peripheral intraconal fat stranding, with no evidence of optic neuritis or dacryoadenitis. The brain MRI with and without intravenous contrast was normal. The chest X-ray was unremarkable. The laboratory studies for systemic and infectious diseases, including antinuclear antibody (ANA), rheumatoid factor (RF), C-reactive protein (CRP), angiotensin-converting enzyme (ACE), antineutrophil cytoplasmic antibodies (ANCA), immunoglobulin G4 (IgG4), and Bartonella antibody, as well as tests for HIV, herpes simplex virus type 2 (HSV 2) IgG, rapid plasma reagin (RPR), QuantiFERON tuberculosis (TB), and purified protein derivative, were all normal or negative. The only abnormal finding was positive HSV 1 IgG titers.

At her two-week follow-up, the patient’s eye redness and pain on palpation had resolved; in addition, her visual acuity remained unchanged, and she had no anterior chamber inflammation or posterior pole involvement. The patient experienced several recurrences during prednisone tapering. Due to her having a history of relapses, methotrexate (12.5 mg subcutaneously, weekly) was added to the treatment regimen. At the time of this writing, the patient remained on the same dose of methotrexate and had successfully tapered prednisone to 0.5 mg daily. Her condition has remained stable, with no inflammation for the past eight months (Figure 1B).

Case 2

A nine-year-old girl was referred to the clinic for evaluation due to concern about a possible rhabdomyosarcoma, OD. For the previous two months, the patient had been experiencing right orbital discomfort and erythema. A month prior to her appointment at our clinic, she had been evaluated by an ophthalmologist who suspected conjunctivitis and prescribed tobramycin dexamethasone drops; however, there was no improvement. She denied experiencing eye pain, vision loss, foreign body sensation, or any other ocular complaints. She had no significant past medical history. Her ocular history included a previous episode of redness in her OS that resolved spontaneously three years prior. Her family history and medical history were unremarkable, as was a review of her systems.

Upon comprehensive ophthalmic evaluation, the patient’s BCVA was 20/25 in the OD and 20/20 in the OS. Intraocular pressure, measured by applanation, was 18 mmHg in the OD and 15 mmHg in the OS. Extraocular movements were full in OU, and confrontation visual fields were intact to count fingers in all quadrants. A slit lamp examination revealed swelling of the right upper eyelid, with a palpable, soft, non-tender mass located in the superonasal area of the conjunctiva and sclera, accompanied by surrounding erythema of the OD. The remainder of the examination of the OD, as well as the examination of the OS examination in its entirety, was normal. A fundus examination showed normal retinae in OU.

**Figure 2:**
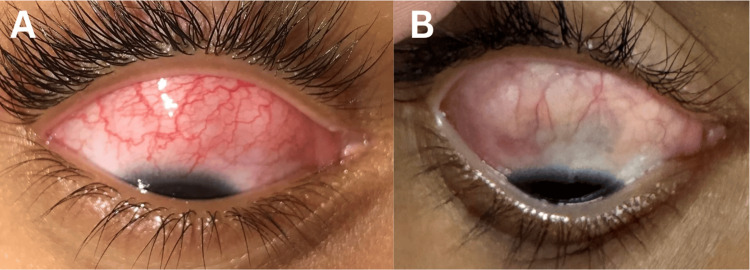
External photographs of the right eye. (A) Downward gaze showing scleral injection. (B) At the 11-month follow-up, downward gaze shows a scleral bluish hue indicative of scleral thinning.

An orbital MRI was ordered to rule out malignancy; imaging revealed normal results, with no masses observed, effectively ruling out rhabdomyosarcoma. However, the patient continued to complain of right orbital discomfort, erythema, and scleral injection, as shown in Figure 2A. Upon examination, notable findings included redness in the OD that did not blanch with phenylephrine 2.5%. An assessment of idiopathic nodular anterior scleritis of the OD was made, and further diagnostic testing was initiated to investigate potential rheumatologic or infectious causes of the scleral inflammation.

Laboratory studies, including a complete blood cell count, a comprehensive metabolic panel, a urinalysis, and a range of specific tests (anti-cyclic citrullinated peptide, HSV 1/2 IgG and IgM, erythrocyte sedimentation rate, ANA, CRP, human leukocyte antigen B27, total complement hemolytic activity, C4, C3, ANCA, RF, HIV 1/2 antibodies, RPR, QuantiFERON-TB Gold, ACE, and lysozyme), all returned results that were either within normal limits or negative. Imaging studies, including a chest X-ray, were also within normal limits.

The initial treatment, based on the patient's weight, consisted of 24 mg of oral prednisolone daily, followed by a gradual taper of 3 mg per week once the active inflammation subsided. However, four months later, the patient experienced a reactivation when the dose was tapered to 6 mg. Prednisolone was increased to 21 mg, and the patient was referred for a rheumatologic evaluation to consider immunomodulatory therapy (IMT). Methotrexate, 15 mg orally, weekly, was eventually initiated. However, 11 months after the initial treatment, the patient continued to have active inflammation despite being on 15 mg of methotrexate and 15 mg of prednisolone daily. She also developed scleral thinning, as depicted in Figure 2B, suggestive of necrotizing scleritis. As a result, biological treatment with adalimumab has been considered.

## Discussion

Anterior scleritis is a relatively rare condition in the pediatric population. The incidence of pediatric scleritis is about 1.2%, with posterior scleritis being the most prevalent form of scleritis in this population [4,5]. Diagnosing this inflammatory disorder in pediatric patients is challenging, largely due to its rarity and the broad spectrum of clinical presentations that can mimic other ocular or systemic conditions. Consequently, the condition is often underdiagnosed or misdiagnosed [7]. This underdiagnosis can lead to a delay in appropriate management, potentially resulting in more severe complications and prolonged discomfort for the patient. Our cases emphasize this diagnostic challenge. Case 1 involved a patient initially treated with antibiotics due to the suspicion that her condition had a bacterial etiology: Her initial presentation included redness, swelling, and pain. However, the lack of antibiotic response led to a re-evaluation and the eventual diagnosis of anterior scleritis. Case 2 involved a patient who was preliminarily evaluated for possible rhabdomyosarcoma. This patient presented with a mass-like effect, raising concerns about a neoplastic process. Further imaging and clinical correlation revealed the inflammatory nature of the condition, confirming the diagnosis of anterior scleritis. In both cases of anterior scleritis, extensive diagnostic workups were performed, including comprehensive laboratory tests, imaging studies, and clinical evaluations. No underlying causes were identified, suggesting an idiopathic etiology for each case.

The appropriate management for scleritis primarily aims to minimize inflammation and prevent damage to adjacent structures. Medical treatment typically includes nonsteroidal anti-inflammatory drugs, corticosteroids, and immunomodulatory agents. In both of the above cases, the patients experienced relapses during steroid tapering, eventually requiring IMT for long-term control and steroid dose reduction. Case 1 remained stable for eight months without any relapses (and remains so at the time of the writing of this case manuscript). In contrast, Case 2, who had necrotizing scleritis, had persistent active inflammation over her 11-month follow-up, despite treatment with steroids and methotrexate, and may require additional IMT for better disease control.

The importance of early recognition and appropriate management cannot be overstated since anterior scleritis in children may be the first manifestation of a systemic autoimmune disease, including granulomatosis with polyangiitis and JIA [5,8]. In several countries, including India, tuberculosis has been found to be a cause of infectious anterior scleritis in children [4], underscoring the importance of further evaluation once a diagnosis of scleritis is made. A comprehensive systemic workup should include testing for autoimmune and infectious disease markers to identify underlying conditions [4,6]. Such an approach ensures timely intervention and the effective management of the disease's ocular and systemic aspects, potentially improving long-term outcomes [4,6].

This report highlights the importance of recognizing this condition in pediatric patients, especially given the atypical presentation that such patients may exhibit. By bringing attention to this kind of case, we aim to enhance awareness and education among healthcare providers, ensuring timely diagnosis and effective management in similar cases.

## Conclusions

Pediatric anterior scleritis is an exceptionally rare condition. In this report, we present two cases of anterior scleritis in young female patients, one of which had necrotized. Both cases were initially misdiagnosed, and both required IMT for long-term management. Despite extensive laboratory workups, no underlying systemic cause was identified in either case, suggesting that each had an idiopathic origin. Given the potential for systemic involvement and serious complications, a multidisciplinary approach is essential to provide comprehensive care. The continued documentation and study of such cases are crucial to ensure timely and appropriate treatment for pediatric patients with this rare condition.
